# Comparing task‐induced psychophysiological responses between persons with stress‐related complaints and healthy controls: A methodological pilot study

**DOI:** 10.1002/hsr2.60

**Published:** 2018-06-26

**Authors:** Elena Smets, Giuseppina Schiavone, Emmanuel Rios Velazquez, Walter De Raedt, Katleen Bogaerts, Ilse Van Diest, Chris Van Hoof

**Affiliations:** ^1^ Electrical Engineering‐ESAT University of Leuven Leuven Belgium; ^2^ Imec Heverlee Belgium; ^3^ Imec, Holst Centre Eindhoven The Netherlands; ^4^ REVAL—Rehabilitation Research Center, Faculty of Medicine and Life Sciences Hasselt University Diepenbeek Belgium; ^5^ Research Group on Health Psychology, Department of Psychology University of Leuven Leuven Belgium

**Keywords:** heart rate, patients, physiology, skin conductance, stress

## Abstract

**Aims:**

Chronic stress is an important factor for a variety of health problems, highlighting the importance of early detection of stress‐related problems. This methodological pilot study investigated whether the physiological response to and recovery from a stress task can differentiate healthy participants and persons with stress‐related complaints.

**Methods and Results:**

Healthy participants (n = 20) and participants with stress‐related complaints (n = 12) participated in a laboratory stress test, which included 3 stress tasks. Three physiological signals were recorded: galvanic skin response (GSR), heart rate (HR), and skin temperature (ST). From these signals, 126 features were extracted, including static (eg, mean) and dynamic (eg, recovery time) features. Unsupervised feature selection reduced the set to 26 features. A logistic regression model was developed for 6 feature sets, analysing single‐parameter and multiparameter models as well as models using recovery vs response‐related features. The highest classification performance (accuracy = 78%) was obtained using the response‐related feature set, including all physiological signals and using GSR‐related features. A worse performance was obtained using single‐signal feature sets based on HR (accuracy = 66%) and ST (accuracy = 59%). Response‐related features outperformed recovery‐related features (accuracy = 63%).

**Conclusion:**

Participants with stress‐related complaints may be differentiated from healthy controls by physiological responses to stress tasks. We aimed to bring attention to new exploratory methodologies; further research is needed to validate and replicate the results on larger populations and patients on different areas along the stress continuum.

## 1 INTRODUCTION

Many studies have revealed the harmful influence of chronic stress on mental and physical health. For example, Stansfeld and Candy^1^ concluded from a meta‐analysis that work stressors are prospective risk factors for common mental health disorders, including depressive and anxiety disorders. Rosengren et al^2^ have shown that psychosocial stressors increase the risk of acute myocardial infarction. Furthermore, associations have been established between psychological stress and depression, cardiovascular disease, and the course of HIV/AIDS.^3^ Another review concluded that both acute and chronic stress research reveals extensive data concerning the stressors' contributions to deteriorated health, including sudden death and myocardial infarction.^4^ Together, these findings highlight the need for affordable and effective early detection of stress problems and preventive interventions of stress‐related mental health disorders.

Stress‐related health problems can be conceptualized into 3 areas along the stress continuum^5^: stress‐related complaints, overstrain, and burnout. A main differentiator between these 3 areas is the chronicity of the complaints. For stress‐related complaints, the time since the onset of the complaints is less than 3 months; for overstrain, more than 3 months; and for burnout, more than 6 months.^5^ Furthermore, persons categorized in the stress‐related complaints group do not yet feel any substantial limitation in their social or professional functioning, whereas this is increasingly the case for both overstrained and burnout patients.^5^

Physiological signals such as heart rate (HR), blood pressure, and galvanic skin response (GSR) have been investigated to detect stress‐related health problems. Studies on autonomous nervous system (re)activity in the context of stress‐related health problems have focused especially on the last stage in the stress continuum, ie, burnout. May et al^6^ found that school burnout was associated with decreased baseline HR variability (HRV). Contradictorily, Morgan et al^7^ showed that persons who score higher on the Maslach Burnout Inventory have significantly higher HRV. De Vente et al^8^ found that burnout patients show higher resting HRs than do healthy controls. Other studies investigating the hypothalamic‐pituitary‐adrenocortical (HPA) activity concluded that burnout patients and controls do not show differences in HPA outcomes.^9^ A review analysis, including 22 studies investigating the physiological mechanisms among burnout patients, concluded that, so far, results are contradictory and inconclusive.^10^ Authors suggest this could be due to differences between studies in the variety and severity of participants' symptoms, co‐morbidity, use of medications, phase in the burnout process, and degree of sick leave.^10^ Although preliminary, such research is promising for the detection of burnout. However, in terms of prevention, it could be valuable to detect stress‐related health problems already in an earlier stage of the stress continuum. To date, no validated questionnaires exist to identify individuals with stress complaints who are vulnerable to develop overstrain and burnout.

In the current study, we, therefore, sought to identify the specific characteristics of persons with stress‐related complaints who are not yet limited in their social or professional functioning, ie, the first stage of the stress continuum. Analogous to previous studies focusing on burnout,^8^ we aimed to investigate the patient's autonomic nervous system responses to and recovery from an acute stressor, as especially these measures may have a great potential for ambulatory stress monitoring and dynamically tailored direct feedback and just‐in‐time behavioural interventions. However, in contrast with most studies in this field, we opted for a less conventional, fundamentally different approach of the data. Traditionally, psychophysiological studies are hypothesis driven, which means that a study is specifically designed to answer a question.^11^ The analysis, therefore, is confirmatory rather than exploratory. However, as technology is continuously improving and wearables become widespread, the amount and nature of psychophysiological data that are available have exponentially grown and call for complementary approaches that allow to maximally explore the wealth of data that are nowadays available. Data scientists have already moved towards more exploratory data‐mining techniques to develop classification algorithms that can unravel new knowledge hidden in the data.^11^ In this methodological study, we will explore and apply this more exploratory approach to analyse the data to evaluate whether persons with stress‐related complaints can be differentiated from healthy participants.

Previous studies have mainly investigated single physiological parameters independently, (eg, Morgan et al,^7^ and De Vente et al^8^), while combinations of multiple physiological parameters and comparisons between single markers could unravel additional insights.^12^ Furthermore, previous studies have focused mainly on static features, ie, the comparison of mean HR in rest and stress tasks. However, both physical fitness and stress research strongly suggests that dynamic features such as response and recovery time can provide additional information regarding physical condition determination.^13^ Based on the research of McEwen,^14^ it was found that failure to shut off allostatic activity after a stress response is one type of allostatic load. This could be reflected in a longer recovery time of the physiological signals after a stressor for patients. It is, therefore, needed to investigate if such dynamic features can also improve the detection of persons with stress‐related complaints.

In this methodological pilot study, we aimed to explore whether a multiparameter classification model that, on the basis of the physiological response to and recovery from 3 standardized laboratory stress tasks, can differentiate between healthy participants and persons with stress‐related complaints. We also assessed which physiological signal(s) is most suitable for the characterization of persons with stress‐related complaints. We included 3 commonly used physiological signals for stress detection, being HR, GSR, and skin temperature (ST). We hypothesized that a classification model combining all 3 physiological signals would outperform models based on the individual signals separately. Furthermore, we compared classification performances on the basis of response and recovery‐related features. We hypothesized, on the basis of the suggestion of Linden et al,^15^ that recovery‐related features could provide additional insight into the difference between healthy participants and persons with stress‐related complaints and, therefore, increase classification performance. Finally, we used both static and dynamic features for classification. We hypothesized, on the basis of earlier findings in physical fitness research,^13^ that dynamic features can improve classification performance. These findings could enhance our understanding of the physiological differences between healthy participants and persons with stress‐related complaints and may advise further strategies to use physiological signals for the early detection of stress‐related health problems.

## 2 METHODS

### 2.1 Participants

A controlled laboratory study was conducted with the approval of the Medical Ethical Committee of the UZ Leuven. All participants signed an informed consent form before participating in the study. In this study, 32 participants, of which 20 healthy participants (10 women, 10 men, mean age = 39.8 y, age range 26‐57 y) and 12 persons with stress‐related complaints (7 women, 5 men, mean age = 38 y, age range 23‐56 y) participated. The focus of this research is on *early* detection of stress‐related health problems; therefore, only persons with stress‐related complaints but without formal diagnosis of any clinical mental health disorder were included.

Healthy participants were recruited in 2 companies in Belgium. They were all employees with a mainly sedentary job who volunteered to participate in the study. They did not receive any compensation for their participation in the study. The healthy participants did not report any physical or psychological disease or complaint, as administered through an intake questionnaire, including, for example, questions related to whether participants suffer of have suffered from psychosis, hyperventilation, depression, epilepsy, panic attacks, and burnout. Persons with stress‐related complaints were recruited at Tumi Therapeutics, a multidisciplinary ambulatory diagnostic and treatment centre that specializes in stress‐related symptoms and syndromes. In return for participation, patients received the psychophysiological diagnostics, which involved the stress tests, free of charge. In addition to the stress test and as part of the standard intake procedure at Tumi Therapeutics, patients also completed a set of questionnaires. Only patients with stress‐related complaints (first phase of the stress continuum) were included. Specifically, the following inclusion criteria were applied: (1) the patient experienced somatic complaints, *and* (2) the complaints started less than 3 months before consultation *and* (3) the patient did not feel limited in his or her personal or professional life, *and* (4) the patient did not suffer from any psychiatric disorder or organic disease. To assess the somatic complaints, the Dutch Symptom Checklist‐90^16^ was used. This questionnaire is often used in clinical practice and research for initial evaluation of patients at intake. The test measures 8 primary symptom levels, ie, sleep difficulties, agoraphobia, hostility, somatization, interpersonal sensitivity, anxiety, cognitive‐performance deficits, and depression. The results can be compared with those of a healthy and clinical norm group for female and male participants separately.^17^ The mean results for the selected patients and normal and clinical norm groups are reported in Table 1. The included patients scored higher on the subscales than did the healthy norm group but scored lower than did the clinical norm group, for all scales, except for somatization and sleep difficulties, for which they scored higher than did the average clinical norm group. The Nijmegen questionnaire for hyperventilation^18^ was used to assess several singular stress complaints such as chest pain, being short of breath, and blurred vision. Included patients scored positive on the Nijmegen questionnaire for hyperventilation, having 18 points or more. All patients confirmed their complaints started less than 3 months before consultation, and all patients were still capable of fully functioning in their social and professional lives. Further, a clinical interview based on the Mini International Neuropsychiatric Interview, which is based on DSM‐IV criteria,^19^, ^20^ was conducted to exclude the existence of any psychiatric disorders. Organic diseases were excluded on the basis of doctor's reports, physical examination, medical tests, and self‐reporting.

**Table 1:** Average scores for male and female patients, compared with a healthy and clinical norm population,^17^ on the different scales of the SCL‐90

SCL‐90 Scale	Male			Female		
	Patients	Healthy Population	Clinical Population	Patients	Healthy Population	Clinical Population
Somatization	25.6 ± 7.7	15	24	32.2 ± 7.7	16	26
Cognitive‐performance deficits	19.8 ± 9	12	20	20.3 ± 9.3	13	21
Interpersonal sensitivity	28.4 ± 7.4	23	35	35 ± 12.6	23	38
Depression	29.6 ± 6.2	18	37	34 ± 7.1	21	44
Anxiety	17.4 ± 3.1	11	23	22 ± 2.6	13	27
Hostility	9.4 ± 2.8	6	10	15.8 ± 7.3	6	10
Agoraphobia	9 ± 2.2	7	11	11.5 ± 4.4	7	12
Sleep difficulties	6.2 ± 1.5	3	5	8.2 ± 3.7	4	7

Abbreviation: SCL‐90, Symptom Checklist‐90.

### 2.2 Procedures

The protocol consisted of 3 stress tests of 2 minutes each: a Stroop Color‐Word test,^21^ a math test, and a stress talk, which were presented using the NeXus 10 MK II software (Mind Media, Herten, The Netherlands). The tasks were given in the same order to all participants. During the Stroop Color‐Word test, colour words were written in an incongruously ink colour; eg, the word red was written in the colour blue. Participants had to respond with the real ink colour, eg, blue in the previous example. During the math test, participants had to successively subtract 7 from the number 1081. To induce additional stress, the experimenter intervened by saying “wrong” or “faster” during the first 2 tasks. During the stress talk, participants had to talk for 2 minutes about a stressful life event. All 3 tests are commonly used to induce stress in laboratory settings.^22^

**Figure 1:**
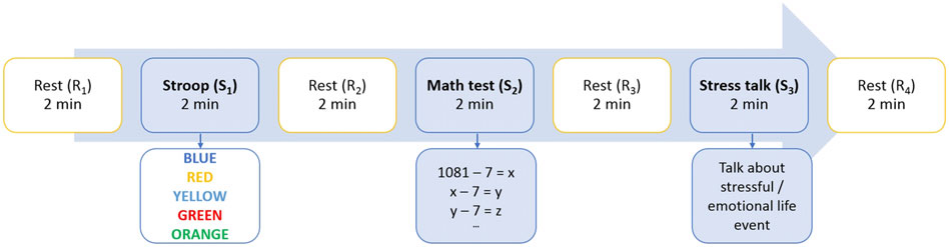
Schematic representation of the experimental protocol. The protocol consisted of 3 stress tasks of 2 min each: a Stroop Color‐Word test, a math test, and a stress talk, separated by rest phases of 2 min

The 3 tests were separated by rest phases of 2 minutes. Before the first and after the last stress test, a resting phase of 2 minutes was included. The timeline of the experiment is shown in Figure 1. For the healthy participants, the protocol additionally included a counting task before the first rest phase and after the last rest phase, as presented in detail by Smets et al.^23^ The goal of this counting task was to control for the physiological response to speaking. We have shown that a stressful task with speech can be distinguished from a nonstressful speaking task, ie, counting.^23^ Since the counting task did not significantly differ from a rest phase, it was removed to reduce the experimental time for the patients. To align the 2 protocols, the 2 counting tasks executed by the healthy participants and the first rest phase executed by both healthy participants and patients were excluded from further analysis.

### 2.3 Measurements

Three physiological signals were measured using the NeXus 10 MK II hardware (Mind Media, Herten, The Netherlands): GSR, ST, and HR. These signals were chosen since they are well‐accepted measures of physiological stress responses.^24^ Additionally, they can easily be implemented in wearable sensors, so that in future research results can be evaluated in daily life settings. Galvanic skin response was recorded at 32 Hz from the distal phalanx of the index and middle fingers of the nondominant hand using Ag/AgCl electrodes embedded in Velcro straps. Skin temperature was recorded at 32 Hz from the distal phalanx of the little finger of the nondominant hand using a thermistor. This is a small point probe, secured by placing tape over the measuring tip to avoid signal contamination by air flow. Heart rate was measured at 128 Hz using a blood volume pulse sensor at the ring finger of the nondominant hand. The sensor used photoplethysmography, which is a light‐based technology to sense the rate of blood flow as controlled by the heart beats. With this signal, instant HR was detected in real‐time by the NeXus software. Participants were asked to keep the hands still, as all signals are susceptible to motion artefacts. Physiological channels were simultaneously streamed to disk and displayed on a PC monitor. Offline, all channels were visually inspected to ensure good quality. There were no missing data. All sensors were attached at least 15 minutes before starting the protocol, allowing the participants to adapt to their position and wearing the equipment.

### 2.4 Feature computation

We applied an exploratory approach towards the signal analysis and feature computation, meaning the outcome for each feature is not hypothesized beforehand but rather explored. Before feature extraction, the physiological signals were standardized with zero mean and unit variance per participant to obtain time series on the same scale. Then, the time series were divided into rest and stress blocks of 2 minutes each, according to the task performed in each segment. This resulted in a total of 7 blocks, 4 rest blocks (R_1_, R_2_, R_3_, and R_4_), and 3 stress blocks (S_1_, S_2_, and S_3_). The first rest block (R_1_) was excluded, since for the healthy participants this task was preceded by a counting task, whereas for the patients, this was the start of the experiment. Next, 2 types of features were calculated: static and dynamic features.

The static features describe the distribution of the physiological signals, eg, the mean and standard deviation, in each block. For each signal, 18 static features were calculated, including the mean and standard deviation, as well as differences of means between pairs of rest or stress blocks (see Table 2). These trends were calculated to explore whether healthy participants and patients differ in the cumulative effect of consecutive stress tasks.

**Table 2:** Overview of the static and dynamic features, calculated for each physiological signal

Feature Name	Blocks	Static or Dynamic	Explanation of Features
Mean	R_2_, R_3_, R_4_, S_1_, S_2_, S_3_	Static	Mean of the physiological signal in the rest/stress block
Standard deviation	R_2_, R_3_, R_4_, S_1_, S_2_, S_3_	Static	Standard deviation of the physiological signal in the rest/stress block
Trend means of stress phases	S_4_ − S_2_, S_4_ − S_3_, S_3_ − S_2_	Static	Difference between the means of different stress phases, eg, S_4_ − S_2_
Trend means of rest phases	R_4_ − R_2_, R_4_ − R_3_, R_3_ − R_2_	Static	Difference between the means of different rest phases, eg, R_4_ − R_2_
Response time	S_1_, S_2_, S_3_	Dynamic	Time in seconds to reach the maximum (HR and GSR)/minimum (ST) starting from the onset of the stress task
Recovery time	S_1_, S_2_, S_3_	Dynamic	Time in seconds to reach the minimum (HR and GSR)/maximum (ST) starting from the onset of the rest phase
Slope	R_2_, R_3_, R_4_, S_1_, S_2_, S_3_	Dynamic	Slope of a straight line fitted through physiological signal in the rest/stress block
Trend response times	S_4_ − S_2_, S_4_ − S_3_, S_3_ − S_2_	Dynamic	Difference between the response times of different stress phases, eg, S_4_ − S_2_
Trend recovery times	R_4_ − R_2_, R_4_ − R_3_, R_3_ − R_2_	Dynamic	Difference between the recovery times of different rest phases, eg, R_4_ − R_2_
Trend slopes of stress phases	S_4_ − S_2_, S_4_ − S_3_, S_3_ − S_2_	Dynamic	Difference between the slopes of different stress phases, eg, S_4_ − S_2_
Trend slopes of rest phases	R_4_ − R_2_, R_4_ − R_3_, R_3_ − R_2_	Dynamic	Difference between the slopes of different rest phases, eg, R_4_ − R_2_

Abbreviations: GSR, galvanic skin response; HR, heart rate; R, rest phase; S, stress task; ST, skin temperature.

**Figure 2:**
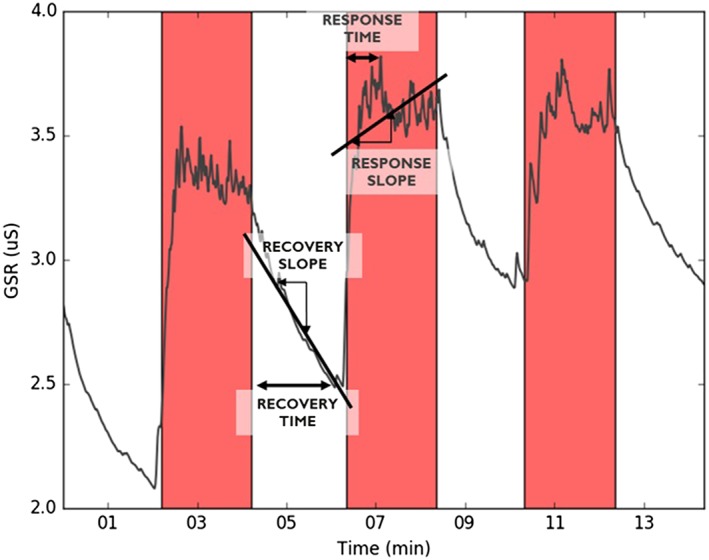
Dynamic feature calculation including recovery time, recovery slope, response time, and response slope. Red bars represent stress phases; white bars, rest. The example signal is galvanic skin response (GSR) from one participant. The same features are calculated for skin temperature and heart rate

The dynamic features represent the transition between different blocks, eg, the transition from rest to stress as response features, and the transition from stress to rest as recovery features. As these features have been shown valuable in physical fitness research,^13^ we investigated whether they can bring additional value for detecting persons with stress‐related complaints. For each signal, 24 dynamic features were calculated. Previous research has indicated that HR and GSR increase^25^ and ST decreases^26^ in response to a stressor. Therefore, for each stress block, the response time was calculated as the time to reach the maximum value for HR and GSR and the minimum value for ST, starting from the onset of the stress task. Similarly, for each rest block, the recovery time was calculated as the time to reach the minimum value for HR and GSR and the maximum value for ST, starting from the onset of the resting phase. Additionally, for all the blocks, a straight line was fitted through the signal, and the slope was calculated. To investigate the cumulative effect of the different stress tasks, the trends across the slopes and the response or recovery times over the different pairs of rest and stress phases were calculated; a positive value for the trend of HR slopes means an increase in steepness of response, while a positive value for the trend of HR response times means an increase in response time after different stress tests. In Figure 2, the GSR to the 3 stress tests, indicated in red, is shown. The recovery time, recovery slope, response time, and response slope are graphically represented. An overview of all the features is presented in Table 2.

### 2.5 Statistical analysis

The goal of this study was to investigate whether healthy participants could be differentiated from persons with stress‐related complaints on the basis of physiological data. Logistic regression (LR) using the Scikit‐learn library of Python 2.7 was used for the analysis.^27^ In LR, the probability of the outcome of the healthy participants versus patients is modelled as a function of the features weighed by coefficients obtained with a training set.^28^

A total of 126 features were calculated. To avoid overfitting, unsupervised feature selection using principal component analysis was applied. We calculated the principal components of the features and selected the number of components, which explained 95% of the variance of the dataset (20 components). Then, we calculated the Pearson correlation of each feature with each principal component and retained the features with a correlation higher than 0.6 with at least one component. This reduced the dataset from 126 to 38 features. Next, to minimize feature redundancy, we calculated the correlation between all features and removed those with a correlation higher than 0.6, reducing the dataset to 26 features.

To compare the classification performance of separate physiological signals and of recovery versus response signals, 6 feature sets were separated on the basis of the reduced feature set: (1) a combination of all features derived from all physiological signals, ie, GSR, HR, and ST (26 features); (2) all features derived from GSR (8 features); (3) all features derived from HR (8 features); (4) all features derived from ST (10 features); (5) all recovery‐related features derived from all physiological signals (13 features); and (6) all response‐related features derived from all physiological signals (13 features).

The performance of each classifier was assessed using a leave‐one‐out cross‐validation. The models were trained on the data of all but one participant and evaluated on the data of this participant; this was repeated until all participants had been evaluated exactly once. This method is often used in different fields of research using small datasets, eg, Healey and Picard^25^ and Woo et al.^29^ To evaluate the model performance specificity, true negative rate (healthy), sensitivity, true positive rate (patient), and accuracy were calculated.

To further investigate the contribution of separate features of different physiological signals to the model, the feature importance was calculated for the model with the highest performance (accuracy). In an LR model, more important features have higher weights. Therefore, the feature importance was calculated by ranking the weights of the model. For the most important features, a *t* test was also performed. For features with significant differences, ie, *P* < .05, the effect size (Cohen *d*) was calculated.^30^ The statistical analyses were performed using the open source SciPy statistical functions library of Python 2.7.

## 3 RESULTS

To evaluate whether physiological data could differentiate healthy controls from persons with stress‐related complaints, classifiers using LR based on 6 feature sets were developed. After unsupervised feature reduction, 26 features were retained: 10 static and 16 dynamic. The accuracy, sensitivity, and specificity for each set are presented in Table 3. The best performance was obtained for the response and GSR feature sets. The worst performance was obtained for the ST and recovery feature set. An intermediate performance was found for the single‐parameter feature set with HR features and feature set with all features.

**Table 3:** Classification performance for each feature set using a logistic regression model^a^

Feature Set	Accuracy	Sensitivity	Specificity
All features	0.72	0.75	0.70
GSR	0.78	0.75	0.80
HR	0.66	0.50	0.75
ST	0.59	0.50	0.65
Recovery	0.63	0.50	0.70
Response	0.78	0.75	0.80

Abbreviations: GSR, galvanic skin response; HR, heart rate; ST, skin temperature.

a The performance is evaluated using accuracy, sensitivity, and specificity. Classifications based on the GSR and response‐related features give the best performance.

**Figure 3:**
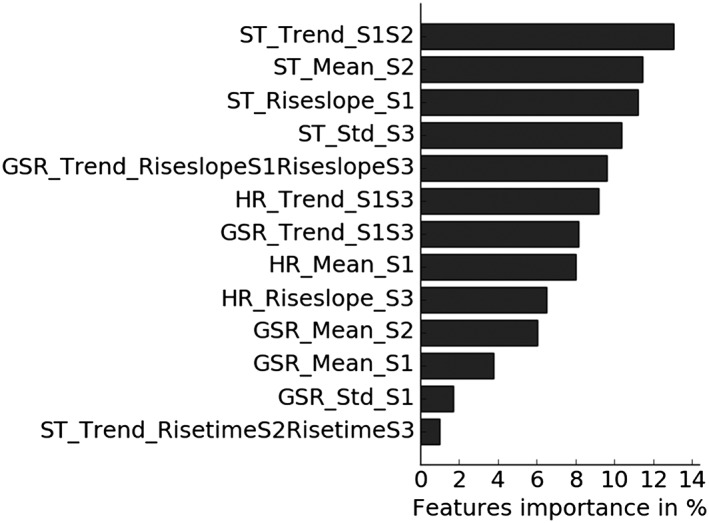
Feature importance of the response feature set based on the relative contribution to the logistic regression model. Feature names contain 3 parts, separated by an underscore: (1) the physiological signal for which the feature was computed, ie, HR, GSR, or ST; (2) the feature (see Table 2); and (3) the stress task(s) for which the feature was computed: S1 = stress task 1 (ie, Stroop Color‐Word test), S2 = stress task 2 (ie, math test), S3 = stress task 3 (ie, stress talk). GSR indicates galvanic skin response; HR, heart rate; ST, skin temperature

For the model based on the response feature set (highest accuracy, including all physiological signals), the relative feature importance was further investigated. Features were ranked on the basis of their relative contributions to the model predictions. The result is shown in Figure 3.

**Figure 4:**
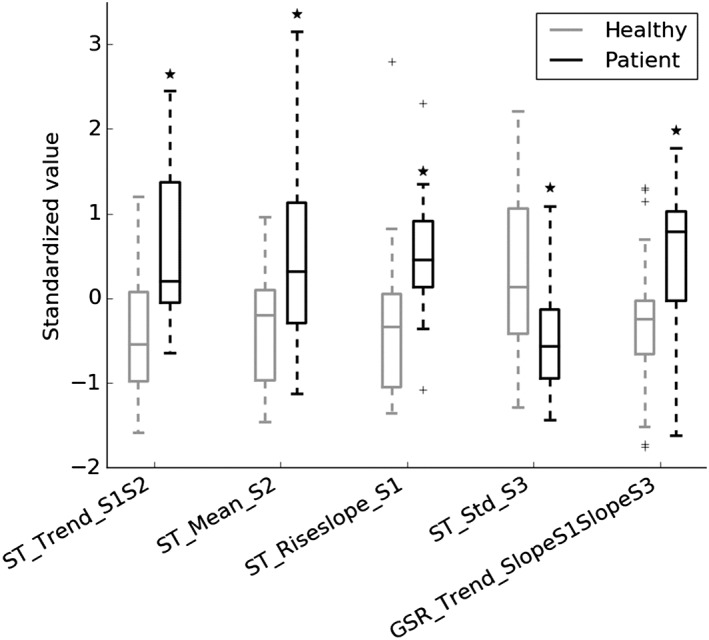
Boxplots of the 5 most important features of the response feature set for healthy participants and patients. Features are represented as standardized values. The boxplot line represents the median, the box extends from the lower to upper quartile values, whiskers extend from minimum to maximum (indicating the range), and flier points (indicated as +) are considered outliers. *P < .05 vs healthy participants, based on a *t* test. Feature names contain 3 parts, separated by an underscore: (1) the physiological signal for which the feature was computed, ie, HR, GSR, or ST; (2) the feature (see Table 2); and (3) the stress task(s) for which the feature was computed: S1 = stress task 1 (ie, Stroop Color‐Word test), S2 = stress task 2 (ie, math test), S3 = stress task 3 (ie, stress talk). GSR indicates galvanic skin response; HR, heart rate; ST, skin temperature

Significant differences for the *t* test and medium to large effect sizes based on Cohen *d* were found for the 5 most important features (others did not show significant differences). These include 4 ST‐ and 1 GSR‐related features. The *t* test was found significant for *P* < .05, and an effect size *d* > 0.5 was considered medium and *d* > 0.8 large.^30^ In Figure 4, the boxplots of these features are shown, comparing the standardized feature values of healthy participants and patients.

The trend of the ST means from the first to second stress task, ie, Stroop test to math test, was close to zero for patients and significantly lower, ie, more negative, for healthy participants (*P* = .007, *d* = 1.06). Since the trend is the difference of S_2_ and S_1_, this indicates that healthy participants have a lower ST in the second stress task compared with the first, while this difference is less distinct for patients. The mean ST from the second stress task, ie, the math test, was significantly higher for patients compared with healthy participants (*P* = .02, *d* = 0.90). The slope of the ST during the first stress task, ie, the Stroop test, was significantly higher for patients compared with healthy participants (*P* = .02, *d* = 0.87). Since both slopes are negative (based on the not normalized values), this indicates a stronger ST decrease for healthy participants. The standard deviation of the ST from the third stress task, ie, the stress talk, was significantly lower for patients compared with healthy participants (*P* = .04, *d* = 0.80). Finally, the trend of the GSR slopes from the first to third stress tasks, ie, Stroop test to stress talk, was significantly higher for patients compared with healthy participants (*P* = .05, *d* = 0.73). This indicates a stronger increase in GSR slopes (ie, a stronger GSR) for patients.

## 4 DISCUSSION

Chronic stress can have a detrimental influence on health, leading, for example, to burnout. To prevent these negative health outcomes, early detection of risk factors is crucial. Stress‐related health problems can be categorized into 3 areas along the stress continuum: stress‐related complaints, overstrain, and burnout. In this pilot study, we investigated the acute physiological response to and recovery from a stress task, for early detection of stress‐related complaints. Persons with stress‐related complaints could be distinguished from healthy participants with an accuracy of 78%, sensitivity of 75%, and specificity of 80%. Whether these results generalize to a larger population, patients with clinical diagnoses such as burnout and chronic fatigue syndrome, or to other types of stressors requires further study.

Our analysis also points to several conclusions with respect to physiological sensing priorities. In previous reports, mainly cardiovascular or GSR features have been used separately as physiological markers of stress‐related diseases (eg, Morgan et al,^7^ and De Vente et al^8^). Our analysis indicated that the best results can be obtained using GSR‐related features. The classification performance using only features related to ST or HR was much lower. However, by combining the response features of HR, GSR, and ST, the performance can be increased, and insights from all physiological signals can be obtained. Furthermore, previous research has suggested that the physiological stress response is person‐dependent, and different participants can show different response levels per physiological signal.^23^, ^31^ For these reasons, and since HR, GSR, and ST are standard measurements readily available in many state‐of‐the art sensors, eg, NeXus 10 MK II, and in multiple wearables such as Empatica E4 (Empatica, Milan, Italy), it is advised to focus further research on the combination of these signals rather than investigate them separately.

Furthermore, when using the recovery‐related features only, as compared with using response‐related features, the accuracy was reduced by 15%. These findings are in disagreement with the suggestion of Linden et al^15^ that recovery features can unravel additional information to distinguish healthy participants and patients. Our findings indicate that these 2 groups differ more in their response to stress than in their recovery from a stress task. A possible explanation could lie in the time frame of the analysis. In our research, the immediate stress response and recovery were analysed in a time frame of 2 minutes during and after the stress task. It is possible that differences become more apparent after a longer period. Further, we focused our research on persons with stress‐related complaints for less than 3 months before consultation. It is possible that if the chronicity of the complaints increases, eg, burnout patients with complaints for more than 6 months, the difference in recovery phase becomes more pronounced. This hypothesis is supported in the meta‐analysis of Miller et al,^32^ who state that when chronic stress first begins, the HPA axis is activated, whereas prolonged chronic stress, which is the case for burnout patients, leads to diminished activity. In a follow‐up study, it could, therefore, be interesting to investigate not only healthy controls versus persons with stress‐related complaints but also persons with stress‐related complaints versus overstrain versus burnout patients. Additionally, it was not possible to compare the response and recovery features to baseline physiology, since the baseline had to be removed owing to the difference in protocol for healthy participants and patients. We suggest that future studies, using this methodology, apply an identical protocol for the 2 groups in order to investigate physiological response and recovery as compared with baseline physiology.

In our analysis, we also investigated which type of features, static or dynamic, is more important for classification purposes. We showed that both types are needed to reach the reported classification performances, with a higher number of dynamic features selected. In previous research towards identification of stress‐related mental health problems, the focus has been on static features. In other research branches, such as the identification of physical condition as opposed to mental, dynamic features have been already incorporated in the analysis (eg, Nishime et al^13^). We suggest that future research in the area of mental and physical health may benefit from including more dynamic features in the analysis. Here, a linear approach was used to calculate the slopes and response and recovery times; in Lim et al,^33^ an exponential approach was also proposed.

Detailed investigation of the most important features for the model based on response‐related features revealed that feature slopes and trends are the most relevant ones (Figure 3). The 5 most important features showed significant differences and medium‐to‐large effect sizes for the healthy participants compared with the patients. A general observation of the results shows that patients often show a more rigid response to stress than did healthy participants (ie, less variation between rest and stress). This could reflect one type of allostatic load, being the inadequate response of the allostatic systems as described by McEwen.^14^ These results highlight the opportunities of using physiological stress responses as a means to discover new insights regarding the process of stress‐related health disorders.

The current study was a methodological pilot study, which was executed in a laboratory setting and with a limited number of patients (n = 12). In the future, a possible application of this methodology could be large‐scale population screenings for early detection of stress‐related health problems. Therefore, to use this methodology in practice, it should be investigated whether similar results can be obtained in real‐life conditions, outside the laboratory. To this end, wearables such as Empatica E4 (Empatica, Milan, Italy) could be used for ambulatory physiological measurement of HR, GSR, and ST. Additional challenges will be related to signal quality.^34^ In the current study, only persons with stress‐related complaints were included. All patients confirmed their complaints started less than 3 months before consultation, and all patients were still capable of fully functioning in their social and professional lives. However, since this information is based on self‐report, it could be incorrect as patients might be unaware of problems in their functioning. Further, we suggest additional research to investigate whether the results generalize to larger populations and patients on different areas along the stress continuum (ie, overstrain and burnout). We aimed with this methodological pilot study to bring attention to new exploratory methodologies; further research is needed to validate and replicate the results.

We conclude that our pilot study demonstrated the potential of physiological signals during the response to a stress task to discriminate healthy participants from persons having stress‐related complaints. Our analysis also showed that a multiparameter classification model based on response‐related features can outperform models based on single parameters (HR and ST) and models based on recovery‐related features only. Investigation of the separate features can provide more insights and enhance our understanding of the physiological differences between healthy participants and persons at risk of stress‐related health problems. Although further research is needed to investigate if these conclusions generalize to a larger population and to multiple clinical diagnoses, these results highlight the potential of using physiological signals and an exploratory approach to gain more insight into the difference between healthy participants and patients. Further longitudinal research using wearable technology to investigate the development of the 3 stages on the stress continuum could provide a powerful technique for better understanding the development of stress‐related disorders. Such research could unravel early detection points for early diagnosis and prevention.

## CONFLICT OF INTEREST

The authors report no competing interests.

## AUTHOR CONTRIBUTIONS

Conceptualization: Elena Smets, Walter De Raedt, Ilse Van Diest, Chris Van Hoof

Data curation: Elena Smets

Formal analysis: Elena Smets, Giuseppina Schiavone, Emmanuel Rios Velazquez

Investigation: Elena Smets, Katleen Bogaerts

Project administration: Elena Smets, Katleen Bogaerts

Resources: Katleen Bogaerts

Supervision: Walter De Raedt, Chris Van Hoof

Writing – original draft preparation: Elena Smets

Writing – review and editing: Elena Smets, Giuseppina Schiavone, Emmanuel Rios Velazquez, Walter De Raedt, Katleen Bogaerts, Ilse Van Diest, Chris Van Hoof
